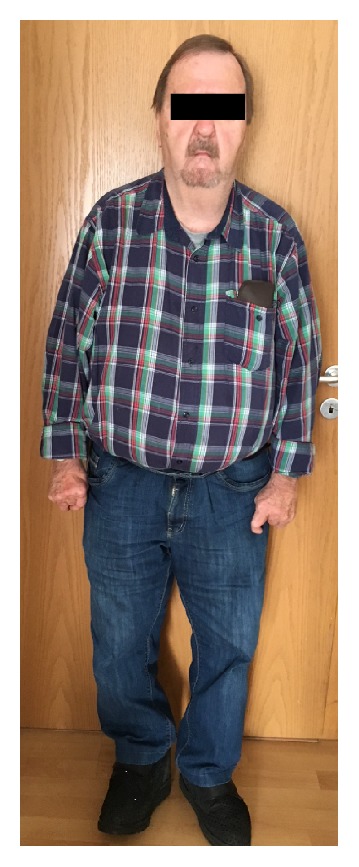# Anesthetic Considerations for an Adult Patient with Freeman-Sheldon Syndrome Undergoing Open Heart Surgery

**DOI:** 10.1155/2018/7862327

**Published:** 2018-02-13

**Authors:** S. Viehmeyer, P. Gabriel, K. Bauer, S. Bauer, R. Sodian, J. N. Hilberath

**Affiliations:** ^1^Department of Anesthesiology and Critical Care Medicine, MediClin Heart Institute Lahr/Baden, Lahr, Germany; ^2^Department of Cardiac Surgery, MediClin Heart Institute Lahr/Baden, Lahr, Germany

## Abstract

Freeman-Sheldon syndrome (FSS) or “whistling face” syndrome is a rare congenital disorder complicated by characteristic facial deformities and muscular contractures. We report on a 64-year-old male patient presenting for surgical replacement of his aortic valve and review the available literature on anesthetic considerations and perioperative management principles. FSS frequently poses a significant challenge to airway management and gaining vascular access. Moreover, these patients are reportedly at risk for developing malignant hyperthermia (MH) or neuroleptic malignant syndrome.

## 1. Introduction

**Figure 1:**
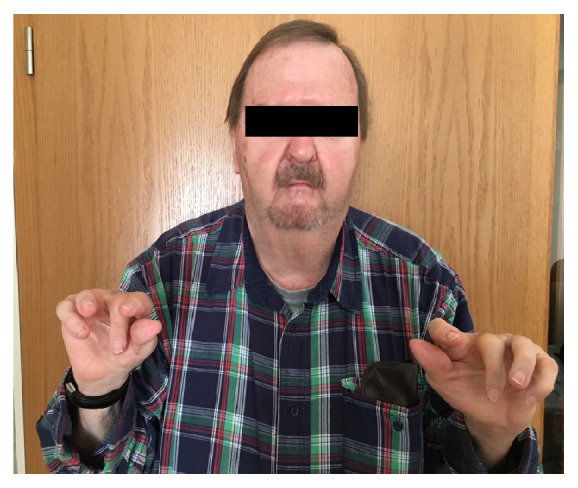


Freeman-Sheldon syndrome (FSS) was first described in 1938 [1] and is part of a group of pathologies referred to as distal arthrogryposis (DA). FSS has been categorized as a specific subtype of DA type 2A in 2006 [2] and is due to a mutation in embryonic myosin, mostly Myosin Heavy Chain 3 (MYH3) [3]. The defect leads to abnormal contraction and relaxation patterns of myocytes frequently already recognized in utero by decreased or absent fetal movement. This mismatch of muscular tonicity directly affects proper skeletal and overall fetal growth, and the facies and distal extremities appear heavily deformed (Figure 1). Patients typically show a distinct physiognomy, initially also described as craniocarpotarsal dysplasia [1, 4]: contractures of musculature and soft tissues lead to characteristic circumoral fibrosis, microstomia, pursed lips, micrognathia, and a short webbed neck with severely limited range of motion. FSS is therefore also described as whistling face syndrome. The distal extremities show malformations like camptodactyly, ulnar deviation, and clubfoot. Kyphoscoliosis and spina bifida occulta can also occur, while strabism and hearing loss are less frequently encountered. Mental retardation is only rarely associated with FSS.

Written consent was provided for publication and photographs of this case.

## 2. Case Report

Our patient was referred from another hospital with newly diagnosed critical aortic valve stenosis (aortic valve area (AVA); 0.7 cm^2^) in combination with a severely decreased left ventricular function (left ventricular ejection fraction (LVEF); 25%). He had a known long-standing history of coronary artery disease and acute coronary syndromes and had undergone coronary stenting procedures repeatedly in recent years. Twice he suffered ST-segment elevation myocardial infarctions (STEMI). Admission chest X-rays showed cardiac congestion with bilateral pulmonary infiltrates. His blood work showed signs of systemic infection including elevated markers of inflammation and leukocytosis. After initiation of antibiotic and heart failure therapy his symptoms improved and he was scheduled for aortic valve replacement. A calculated EuroSCORE II of 2.08% lead to our center's interdisciplinary heart team decision for surgical aortic valve replacement (SAVR).

His past medical history was otherwise significant for type 2 diabetes, colonic diverticulosis, and COPD. During childhood and adolescence he had undergone numerous surgical procedures to correct skeletal deformities. Despite significant physical disabilities the patient had remained ambulatory and able to sufficiently perform activities of daily living (ADL) without assistance (Figure 2).

## 3. Anesthetic Management

The patient did not report problems with previous anesthetics on preoperative evaluation. Written records of previous anesthetics could not be obtained. Given his orofacial anatomy including microstomia, retrognathia, and limited mobility of his neck, his airway was secured via awake nasal fiberoptic intubation (AFOI) (Figure 1). After local anesthesia of his naso- and oropharynx with aerosolized lidocaine (4%) and decongestant treatment of the nasal mucosa (xylometazoline 1%), he received small incremental doses of sufentanil (total dose 35 *μ*g) for additional analgesia and light sedation during intubation. Endotracheal intubation was successful without distress or hypoxemic events. After induction of general anesthesia (GA), direct laryngoscopy confirmed a Cormack Lehane 4 view. As alternate plan of securing the airway in case of failed AFOI or difficulties with ventilation, the attending surgeon remained in standby during induction to perform an awake tracheotomy in the spontaneously breathing patient.

Prior to skin incision, cisatracurium (10 mg) was administered once. GA was maintained by propofol (4-5 mg/kg/h) and sufentanil (50–70 *μ*g/h) infusions titrated to bispectral indices between 40 and 60. A trigger-free anesthetic was chosen to mitigate the risk for malignant hyperthermia (MH) or anesthesia-induced rhabdomyolysis.

After uneventful replacement of his aortic valve with a 23 mm bioprosthesis (cross clamp time 56 min, cardiopulmonary bypass time 72 min) the patient was transferred to our intensive care unit sedated and intubated.

Sedation was stopped on postoperative day (POD) 1. The patient initially presented disoriented with weak muscular tone and only minimal movement. During a spontaneous breathing trial at that time his respiratory mechanics appeared insufficient and labored and he was lacking sufficient cough and appropriate swallowing reflexes. Subsequently, light sedation with propofol was continued (Richmond Agitation Sedation Score- (RASS-) 1). Six hours later, the patient's sensorium and muscular tone had recovered enough to allow for safe extubation.

During his postoperative course, creatine kinase levels were repeatedly measured and remained in low-normal range. Also, the patient never developed fever or acidemia.

The patient was discharged from the ICU on POD 4 and left the hospital to rehabilitation on POD 7.

## 4. Discussion

To our knowledge this is the first report on the anesthetic management of an adult FSS patient undergoing cardiac surgery.

While FSS remains a rare condition, the likelihood of perioperatively caring for adult patients with congenital pathologies will increase in the future. With an increasing life expectancy, ailments like cardiovascular disease become more prevalent and might require (surgical) interventions.

Managing orphan diseases and congenital syndromes remain challenging. Most available literature to guide decision-making stems from pediatric patients. The fundamental topics for perioperative clinicians caring for FSS patients are the management of a difficult airway and vascular access [5]. Moreover, pharmacologic choices in patients at risk for MH and additional perioperative complications require heightened vigilance within the care team.

Even though difficult airway anatomy is frequently encountered in patients undergoing thoracic surgery, patients with FSS almost invariably present with a challenging anatomy. Their small mouth opening and receding chin make oral intubation difficult and the limited nasopharyngeal space might render nasal placement of an adequately sized endotracheal tube impossible. A laryngeal mask airway can be a viable alternative in some patients without significant reflux disease or impaired gastrointestinal motility. AFOI is deemed best practice to secure these patients' airway. However, AFOI in FSS frequently can present a significant challenge, even for experienced practitioners (Figure 1).

Establishing venous and arterial access can also be difficult in patients with contractures. The widespread utilization of ultrasound to visualize vessels has significantly improved success rates of vascular cannulation [6] (Figures 1 and 2).

While regional and local anesthesia are considered ideal in these patients and recommended where possible [7] they are not an option for cardiac surgery.

The choice of sedative and anesthetic drugs is still debated notwithstanding that detrimental side effects seem to be rare. Benzodiazepines have been safely used as premedication although their intrinsic potential for muscle relaxation must be taken into account [4, 8]. Ketamine or small doses of short-acting opioids might present viable alternatives. Given a shortage of remifentanil in Germany, we chose sufentanil as primary analgesic and sedative. In our patient benzodiazepines were omitted to avoid additional muscular weakness and prolonged postoperative mechanical ventilation and recovery. Muscle relaxation was, however, considered necessary by the surgical team to improve exposure. We chose a single dose of cisatracurium, which is degraded by Hofmann elimination independent of cholinesterase activity or metabolism and without metabolites with intrinsic relaxant activity. Nevertheless, our patient showed significantly slowed recovery and safe extubation was only possible with delay.

Several cases of hyperthermia possibly related to the use of anesthetics have been reported in FSS, and an inherent risk of MH cannot be safely confirmed or ruled out to date [9, 10]. Therefore, some authors recommend avoiding the use of potential triggers as best practice in FSS altogether. However, volatile agents as well as other known trigger substances have been used in pediatric patients: uneventful inhalational induction and maintenance of anesthesia have been described with sevoflurane [2, 8] whereas halothane has been linked to hyperpyrexia in several cases [5]. Muscular rigidity as an early sign of MH has been described using halothane plus succinylcholine [2]. However, in other cases, halothane has also been found not to be harmful [7, 11]. Importantly, all proven or suspected cases of MH were successfully treated with dantrolene. Given these nonuniform recommendations in the literature we used a trigger-free setup to avoid any risk of MH. For induction, maintenance, and postoperative sedation a combination of propofol and short-acting remifentanil would have been our preference. Unfortunately, remifentanil was not available in Germany at the time. We therefore decided on sufentanil as intraoperative opioid given its predictable context-sensitive half-life. Still, the prolonged weaning and muscular weakness in our patient highlight the need for increased perioperative vigilance despite careful titration of well-controllable anesthesia drugs. After extubation we preferentially used NSAIDS and carefully titrated piritramide, a selective *μ*-receptor agonist, based on the visualized analogue pain scale.

Metoclopramide has been linked to neuroleptic malignant hyperthermia in one patient with FSS, which was terminated by dantrolene [9]. Given several other case reports on neuroleptic malignant syndromes and hyperthermia we recommend avoidance of atypical neuroleptics altogether. For most indications prompting their use like postoperative nausea and vomiting (PONV), perioperative delirium, or postoperative delayed gastric emptying there are adequate alternatives. The use of alpha-2 agonists seems also a rational choice in these patients even though no validated studies are available to date.

## 5. Conclusions

Adult FSS patients undergoing cardiac surgery can be safely managed. Advance interdisciplinary planning and assignment of appropriate resources to the management of a potentially challenging airway and vascular access anatomy as well as planning for a prolonged ICU stay enable safe patient outcomes. A well-balanced, trigger-free anesthetic with short-acting opioids, limited use of muscle relaxants, and postoperative sedatives as well as neuroleptic drugs seem prudent in FSS.

## Figures and Tables

**Figure 2:**